# Editorial: GnRH agonist triggering of final oocyte maturation: Improving safety without compromising success

**DOI:** 10.3389/fendo.2023.1171974

**Published:** 2023-03-16

**Authors:** Johnny Awwad, Juan Garcia-Velasco, Peter Humaidan

**Affiliations:** ^1^Faculty of Medicine, American University of Beirut, Beirut, Lebanon; ^2^Division of Reproductive Medicine, Sidra Medicine, Doha, Qatar; ^3^IVI Madrid - Clínica de Reproducción Asistida y Fertilidad (IVIRMA), Madrid, Spain; ^4^Division of Reproductive Endocrinology, Aarhus University, Aarhus, Denmark

**Keywords:** GnRH agonist, oocyte maturation trigger, progestin-primed ovarian stimulation, OHSS, assisted reproduction (ART)

The use of GnRH agonists to trigger final oocyte maturation in assisted reproduction has gained momentum over the past decade because of their safety records in mitigating the risk for severe OHSS. While the shorter agonist-induced LH surge and the earlier demise of the corpus luteum are significant contributors to the safety profile of agonist triggering agents, they appear to compromise embryo sustained implantation when used with conventional luteal supplementation protocols

Dissociating the follicular and luteal events through cycle segmentation has therefore become the preferred method for GnRHa triggering, namely for clinical applications such as preimplantation genetics, gamete/embryo cryopreservation and egg donation. This growing trend was facilitated largely by the significant breakthrough in cryobiology and the successful outcomes of embryo vitrification.

When a fresh embryo transfer is planned, alternative strategies to improve the profoundly deficient luteal phase are suggested. The use of intensive hormonal supplementation has been inadequately investigated. Whether standardized or individualized protocols are more suitable remain inconclusive. The administration of low dose hCG to prevent corpus luteum demise was found to be successful in restoring reproductive outcomes. These protocols nonetheless are very heterogenous and differ in the dose and timing of hCG administration. Their safety when used in high responders require further confirmation.

In their minireview, Najdecki et al. discuss the usefulness of gonadotropin-releasing agonists in oocyte donation programs. The authors present evidence from the medical literature supporting the beneficial effects of agonist triggering protocols in reducing the risks for severe OHSS. The risk nonetheless is not completely eliminated, which prompts extreme caution during stimulation of otherwise healthy women donors. They also discuss the role of serum LH levels measured at various stages of the ovarian stimulation cycles to predict and mitigate the risks for empty follicle syndrome.

Using a retrospective study design, Kalafat et al. evaluated the clinical performance of a GnRH agonist triggering when used with a progestin-primed versus GnRH antagonist suppressed protocol. While the pituitary suppressive effects of progestins are believed to induce profound LH depletion (1), the authors observed GnRHa-induced LH surges with paradoxically steeper rises and higher magnitudes in association with progestin-primed cycles compared with antagonist cycles. While such observation requires to be validated by well designed prospective studies, the implications may be interesting because of the opportunity to use readily available alternatives to GnRH antagonists with lower costs and potentially higher efficacy. These findings also undermine the previous belief that progestin pituitary suppressive effects are mediated centrally through LH receptor depletion.

The prospective observational study by Martazanova et al. lends important information on luteal hormonal levels following various oocyte maturation triggering protocols in high responders. Serum LH, estradiol, and progesterone levels were measured and compared following GnRHa trigger with hCG rescue dose (1,500 IU) on the day of oocyte pick-up vs dual trigger (GnRHa + 1,500 IU hCG) vs conventional 10,000 IU hCG. Progesterone levels measured on ovum pick-up (OPU) day 5 were significantly lower when small dose hCG (1,500 IU) was administered on the day of GnRH agonist trigger compared to hCG rescue in the day of OPU. Furthermore, progesterone levels on the day of OPU were significantly higher in the non-pregnant group. While the use of mini hCG dose enhanced significantly clinical outcomes in high responders undergoing fresh embryo transfers, it was also associated with a risk of OHSS similar to conventional hCG.

In the last study, Merkison et al. discuss the optimization of fertility preservation protocols in women with inherited hematological disorders undergoing fertility-threatening procedures. The investigators present data from the medical literature supporting the effectiveness and safety of GnRH agonist triggering protocols for women presenting for fertility preservation.

These interesting manuscripts provide further evidence for the benefits of the paradigm shift – agonist trigger to induce final oocyte maturation - while revealing gaps that still require further research. The fact that OHSS is almost non-existent today is the clinical proof that agonist trigger is clearly a huge step forward to improve the care we provide to our patients. Still, further fine tuning is needed for individualized luteal phase support.

## Author contributions

All authors listed have made a substantial, direct, and intellectual contribution to the work and approved it for publication.
